# Nutritional status of congenital heart disease (CHD) patients: Burden and determinant of malnutrition at university of Nigeria teaching hospital Ituku – Ozalla, Enugu

**DOI:** 10.12669/pjms.315.6837

**Published:** 2015

**Authors:** Ijeoma Arodiwe, Josephat Chinawa, Fortune Ujunwa, Dabere Adiele, Mildred Ukoha, Egbuna Obidike

**Affiliations:** 1 Dr. Ijeoma Arodiwe, MBBS, FMCPaed. Consultant Pediatrician, Department of Pediatrics, University of Nigeria Teaching Hospital (UNTH), Ituku- Ozalla, Enugu State, Nigeria; 2 Dr. Josephat Chinawa, MBBS, FMCPaed. Lecturer, Department of Pediatrics, College of Medicine, University of Nigeria, University of Nigeria Teaching Hospital (UNTH), Ituku- Ozalla, Enugu State, Nigeria; 3 Dr. Fortune Ujunwa, MBBS, FMCPaed. Consultant Pediatrician, Department of Pediatrics, University of Nigeria Teaching Hospital (UNTH), Ituku- Ozalla, Enugu State, Nigeria; 4 Dr. Dabere Adiele, MBBS, FWACP. Consultant Pediatrician, Department of Pediatrics, University of Nigeria Teaching Hospital (UNTH), Ituku- Ozalla, Enugu State, Nigeria; 5 Dr. Mildred Ukoha, MBBS, FWCP. Consultant Pediatrician, Department of Pediatrics, Enugu State University Teaching Hospirtal, Enugu, Nigeria; 6 Prof. Egbuna Obidike, MBBS, FMCPaed. Professor, Department of Pediatrics, College of Medicine, University of Nigeria, University of Nigeria Teaching Hospital (UNTH), Ituku- Ozalla, Enugu State, Nigeria

**Keywords:** Congenital heart disease, Malnutrition, Prevalence, Determinant

## Abstract

**Background and Objectives:**

Children with congenital heart disease (CHD) are prone to malnutrition. This can have a significant effect on the outcome of surgery. Our objective was to determine the burden and determinant of malnutrition in children with several types of congenital heart disease (CHD).

**Methods:**

This is a descriptive cross sectional study of children attending the outpatient clinic of UNTH, Ituku – Ozalla, Enugu State, over a six year period from March 2007 to April 2014. Data analysis was done with Statistical Package for Social Sciences (SPSS) version 19 (Chicago IL).

**Results:**

Forty thousand one hundred and twenty three (40,123) children attended the outpatient clinic during the study period. Of these, 50 had congenital heart disease, from which 46 were found to have various degree of malnutrition, giving a prevalence of 92% among children with congenital disease and 0.11% in the general population. Malnutrition showed significant correlation between age in years, age appropriate dietary adequacy and pulmonary hypertension. (r= 0.22, p = 0.01; r = 0.20, p = 0.02; r = 0.15, p = 0.01).

**Conclusion:**

Children with CHD develop severe malnutrition and growth failure. The significant contributing factors are mean age at presentation and age appropriate dietary adequacy.

## INTRODUCTION

Malnutrition poses a burden not only on the health system, but the entire socio-cultural aspect of the country.^1^ Malnutrition can be defined as a state of nutrition where the weight for age, height for age and weight for height indices are below -2 Z-score of the NCHS reference.^2^ It constitutes a major public health problem in developing world and serves as the most important risk factor for the burden of disease especially among children.

Malnutrition occurs among children with congenital heart disease, irrespective of the nature of the cardiac defect and the presence or absence of cyanosis^3^ Children with congenital heart disease (CHD) are prone to malnutrition for several reasons including decreased energy intake, increased energy requirements, or both.^4^ For instance, children with malnutrition due to ventricular septal defects (VSD) have been shown to have a 40% elevation in total energy expenditure (TEE). Children with CHD in which there is congestive heart failure or an increase in after load (coarctation of the aorta or pulmonary hypertension) often present with increased energy expenditure.^5^,^6^ This is because the heart must work much harder in order to pump an adequate blood for body metabolism. Another reason for the increased metabolic rate seen in children with CHD is due to their body composition. This is because decreased caloric intake and greater energy expenditure will make less energy available for fat deposition. As a result, they will have an elevated percentage of lean body mass which tends to increase their basal metabolic rate.^7^

Chronic hypoxaemia also affects growth by inducing anorexia which causes inefficient processing of nutrients at the cellular level.^4^ Other factors that can cause malnutrition in children with congenital heart disease is pulmonary hypertension, and age of children at presentation.^8^,^9^

Assessment of nutritional status among children with congenital heart disease (CHD) is a very important issue often neglected in pediatrics practice and in Nigeria, its importance therefore cannot be overemphasized. This study therefore was aimed at determining the burden and determinant of malnutrition in children with several types of congenital heart disease (CHD). The results will help us to improve on nutritional counseling and early surgical correction to optimize the growth of children with CHD. This will also enable us to establish a baseline data where other related issue will hinge on. We are not aware of any study of this nature from this environment.

## METHODS

This is a descriptive cross sectional study of children attending the outpatient clinic of UNTH, Ituku – Ozalla, Enugu State, over a six year period (March 2007 to April 2014). The prevalence of malnutrition and it contributory factors was determined in 50 patients with CHD using information derived from their medical record and an interview with the parents / caregiver using a semi - structured researcher administrated questionnaire. Sixty five children presented with congenital heart disease from 2007 till the time of study. However only 50 had complete data especially echocardiography results.

They were grouped according to cardiac diagnosis: group APAH (n = 22), acyanotic patients with pulmonary hypertension; group AWTPAH (n = 14), acyanotic patients without pulmonary hypertension; group CWTPAH (n=12), cyanotic patients without pulmonary hypertension; and group CPAH (n = 2), cyanotic patients with pulmonary hypertension.

Information on socioeconomic level; using the Oyedeji’s^10^ classification, birth weight and nutrition history, number of siblings, were obtained through interviews with parents/care givers and older children. There serum protein and heamoglobin estimation were done.

Standardized measurements of weight, length, and head circumference were taken by one nurse Malnutrition was classified as mild, moderate, or severe when patient weight was 80–90%, 70–79%, and < 70% of ideal weight for length % which is same as < -1 to > -2 Z score, < -2 t0 Z score > -3, and < -3 Z score respectively, based on the WHO standard.

### Ethical clearance

Ethical approval for the study was obtained from the Research and Ethical Committee of the University of Nigeria Teaching Hospital Ituku Ozalla. Informed consent was sought from parents or care givers of potential subjects and controls before enrolling them into the study.

### Data Analysis

Data analysis was with Statistical Package for Social Sciences (SPSS) version 19 (Chicago IL). Chi-square test was used to test for significant association of the proportion. A p-value of < 0.5 was regarded as significant. Multivariate correlation of independent variables and Stepwise multiple linear regressions were used to determine factors that correlates with malnutrition in the subjects.

## RESULTS

Forty thousand one hundred and twenty three (40,123) children attended the outpatient clinic during the study period, of these, 50 had congenital heart disease, from which 46 were found to have various degree of malnutrition, giving a prevalence of 92%. Severe malnutrition contributed a whopping 60% of the total. The mean age was 4.66 ± 5. 02 years with range of 1month to 16 years. Out of the 50 cases 31(62.0%) were male and 19 (38.0%) female, giving a male to female ratio of 1.6:1. Acyanotic patients with pulmonary arterial hypertension constitute the largest group 22(44%) and VSD was the most common defect 12 (24%) in that group. Table-I shows the frequency of cardiac diagnosis. There was significant difference between groups and the degree of malnutrition. (p < 0.05). Table-II

**Table-I:** Shows the frequency of Cardiac diagnosis.

Diagnosis (n)	Frequency (%)
Acyanotic patients without pulmonary hypertension	14(28)
ASD + VSD	2(4)
ASD	4(8)
VSD	7(14)
AR + AS	1(2)
Acyanotic patients with pulmonary hypertension	22(44)
ASD + PDA + VSD	1(2)
VSD + PDA	5(10)
MR + MS	1(2)
VSD	12(24)
PDA	3(6)
Cyanotic patients without pulmonary hypertension	12(24)
TOF	8(16)
DORV + PS	2(4)
EBSTEINS ABNORMALY	2(4)
Cyanotic patients with pulmonary hypertension	2(4)
TRUNCUS ARTERIOSUS TYPE A + VSD	2(4)

NB: VSD, ventricular septal defect; PDA, patent ductus arteriosus; AS, aortic stenosis; AR, aortic regurgitation; MS, mitral stenosis; MR, mitral regurgitation; ASD, atrial septal defect; TOF, tetralogy of Fallot; PS, pulmonary stenosis; DORV, double outlet right ventricle.

**Table-II:** Shows selected characteristics of children enrolled in the study by their groups.

Group						
Characteristics	APAH (n=22)	AWTPAH (n = 14)	p – value	CPAH (n = 2)	CWTPAH (n=12)	
*Sex:*						
Male	14	8	0.21	1	8	0.06
Female	8	6		1	4	
Age in years	8.06(5.64)	3.95 (4.51)	0.01*	9.32(3.11)	5.5(5.32)	0.04*
Weight (kg)	9.35(10.84)	14.24(10.01)	0.02*	13.83 (8.03)	18.03(3.22)	0.01*
Height (cm)	87.57(46.39)	120.55(37.27)	0.03*	112.11(10.10)	88.3 (34.38)	0.02*
Weight for height %	62(8.00)	82(18.0)	0.02*	70(5.00)	79.0(6.00)	0.04*
Weight- for- height z score	-3.83 ± 1.65	-2.76 ± 0.22	0.01*	-3.56 ± 0.53	-2.23 ± 0.35	0.04*
Total protein (g/L)	61.47(5.36)	57.38(10.07)	0.05	60.32(10.9)	61.00(4.08)	0.15
Heamoglobin (g/dl)	18.34(5.66)	11.5(4.3)	0.02*	29.10 (5.5)	25.25(8.23)	0.01*
Mean pulmonary artery pressure (mm Hg)	50.1(10.9)	10.9(5.6)	0.01*	60.83(17.8)	20.8(5.0)	0.01*
Saturation of oxygen %	80.8(3.24)	95. 01(2.2)	0.01*	61.6(12.0)	65.6 (15.0)	0.07
Triceps skin fold (mm)	5.60(1.10)	6.05(4.10)	0.05	4.01(2.01)	6.23 (3.70)	0.06
Subscapular skin fold (mm)	4.05(0.48)	5.80(2.90)	0.07	5.01(2.08)	4.90 (2.70)	0.05

NB: Figures shown are mean ± one standard deviation (±1SD) of the mean

* Statistically significant.; Group APAH (n = 22), acyanotic patients with pulmonary hypertension;

Group AWTPAH (n = 14), acyanotic patients without pulmonary hypertension; Group CWTPAH (n=12), cyanotic patients without pulmonary hypertension; and Group CPAH (n = 2), cyanotic patients with pulmonary hypertension.

Mild malnutrition was seen in acyanotic group without pulmonary hypertension but most cyanotic group patients were in severe malnutrition state, and stunting was more common than wasting among cyanotic group. Table-II.

Table-III shows the correlation of the independent variable on malnutrition. Malnutrition showed significant correlation between age in years, age appropriate dietary adequacy and pulmonary hypertension. (r= 0.22, p = 0.01; r = 0.20, p = 0.02; r = 0.15, p = 0.01)

**Table-III:** Multivariate correlation of independent variables with malnutrition.

	Normal nutrition (n = 4)		Malnutrition (n=46)	
Independent variable	Correlation coefficient (r)	p – value	Correlation coefficient (r)	p – value
Age (years)	0.32	0.43	0.22	0.01*
Age appropriate dietary adequacy	0.19	0.31	0.20	0.02*
Family size	0.45	0.15	0.32	0.21
Socioeconomic class	- 0.29	0.12	-0.30	0.45
Pulmonary hypertension (mmHg)	- 0.38	0. 51	0.15	0.01*
Oxygen saturation (%)	- 0.33	0.08	-0.35	0.24

**Table-IV:** Shows the frequency of malnutrition by Cardiac diagnosis groups of Cardiac diagnosis.

Nutritional Classes	AWTPAH (n = 14)	APAH (n - 22)	X^2^ (p-value)	CPAH (n - 2)	CWTPAH (n = 12)	%	X^2^ (p – value)
Normal	2	2		0	0	8%	
Mild	4	4	12.69(0.06)	0	1	18%	7.95(0.03*)
Moderate	2	2	9,78(0.05)	0	3	14%	8.30(0.04*)
Severe	6	14	4.34(0.01*)	2	8	60%	2.78(0.01*)
% malnutrition by groups	26%	43%		4.3%	26%		

X^2^: chi square.

Table-V shows a stepwise multiple linear regressions of factors that correlate significantly with malnutrition in the subjects. It shows age in years, degree of dietary adequacy and pulmonary hypertension were the best contributors to malnutrition in the subjects. (r = 0.396, t = 2.186, p = 0.038; r = -0.212, t = 1.170, p = 0.025; r = 0.634, t = 1.231, p = 0.001).

**Table-V:** Stepwise multiple linear regressions of factors that correlates with malnutrition^a^ in the subjects.

Model	Unstandardized Coefficients		Standardized Coefficients(r)	T	p-value	95% Confidence Interval for B	
	B	Std. Error	Beta			B	Std. Error
(Constant)	1.282	277		4.627	0.000	0.714	1.851
Age (years)	0.005	0.051	-.212	1.170	0.025*	-0.015	0.004
Dietary adequacy	0.034	0.016	0.396	2.186	0.038*	0.002	0.066
Pulmonary hypertension	0.021	0.010	0.634	1.231	0.001*	-0.003	0.034

a Dependent Variable: Malnutrition,

* Significant.

There was a significant difference in mean age at presentation, appropriate dietary adequacy pulmonary hypertension and malnutrition. Other sociodemographic variables did not show any significant difference.

## DISCUSSION

The above study had shown that children with CHD are likely to develop severe malnutrition and growth failure. The most important potent trigger of severe malnutrition among children with congenital heart disease (though more in cyanotic heart disease) is pulmonary hypertension. This finding is in keeping with the study of Pitmann et al.^11^, they also noted the impact of pulmonary hypertension on growth and nutrition in cyanotic and acynotic heart disease, and found that cyanotic patients with pulmonary hypertension were more affected than acynotic children with hypertension. The preferred reason for this pulmonary hypertension induced malnutrition among children with cyanotic heart disease could be compensated metabolic acidosis caused by hypoxia. In addition, chronic hypoxia, anorexia and inefficient processing of nutrients at the cellular level are also implicated.^12^

Children with cyanosis and pulmonary hypertension had severe malnutrition and were stunted while those with acynotic heart disease were wasted. Birgül and colleagues^13^ also noted stunting to be more common than wasting in cyanotic heart disease. They also investigated the impact of pulmonary hypertension on growth and nutrition in cyanotic heart disease, and found that cyanotic patients with pulmonary hypertension were the most severely affected group.^13^

Another important factor that cause malnutrition among children with congenital heart disease in this study is dietary inadequacy. This has a negative effect on nutrition and growth of children with CHD. This they do by inducing Chronic hypoxia, furthermore, malabsorption and maldigestion are thought to play a role in cardiac cachexia due to dietary inadequacy.^14^ Unfortunately we did not assess the effect of malabsorption on cardiac disease in our patients.

We obtained a strong correlation between age of presentation and malnutrition among our subjects. Children in the older age group are more prone to malnutrition and poor growth. This finding was corroborated by Venugopalon et al.^15^ Possible reasons for this may be due to early surgical intervention for their heart disease at early age in his study, while there was delay in surgical intervention in our patients which made the long term impact of the disease more pronounced.

Another predictor of malnutrition among children with congenital heart disease in this study is weight for height using z-score. Children with cyanotic heart disease with hypertension had the lowest z-score (stunting). This is in agreement with our earlier report that Children with cyanosis and pulmonary hypertension had severe malnutrition and were stunted. Currently, the WHO recommended the use Z-Score or SD system to grade under nutrition.^15^ This method measures all the three indices and expresses the results in terms of Z scores or standard deviation units which other methods don’t.^16^

We also used subscapular skin fold to measure malnutrition among these children, the decrease in the value of the subscapular skinfold increases the probability of the occurrence of the immediate or acute types of malnutrition.^17^ The subscapular skinfold denotes a good correlation with total body fat, and the quantity of fat deposited in the trunk region provides support for the early detection of malnutrition and obesity.^17^ Following this, in congenital heart disease, the presence of energy imbalance, the feeding problems and an increased metabolic rate resulting from poor cardiac function may lead to smaller reserves of central adiposity, with congenital heart disease being considered a predictive factor for malnutrition.

Finally, the overall prevalence of malnutrition among children with congenital heart disease is 92%. This is similar to the work of Okoroma et al.^18^ in Lagos and Cameroon^19^ elsewhere where a prevalence of 90.4% and 97% were obtained respectively.

## CONCLUSION

Children with CHD develop severe malnutrition and growth failure. The significant contributing factors are mean age at presentation and age appropriate dietary adequacy. However pulmonary hypertension appear to be the most important factor, and cyanotic patients with pulmonary hypertension are the ones most severely affected.

### Recommendation

We recommend routine nutritional rehabilitation as standard practice in the management protocol of these children and early surgical repair / interventional procedure.
